# Oral Health Status of Physically Disabled Inpatients – Results from a Hungarian Single-Centre Cross-Sectional Study

**DOI:** 10.3290/j.ohpd.b2448609

**Published:** 2021-12-18

**Authors:** Mercédesz Orsós, Júlia Moldvai, Fanni Simon, Miklós Putz, Gergő Merész, Orsolya Németh

**Affiliations:** a Assistant Lecturer, Department of Community Dentistry, Semmelweis University, Budapest, Hungary. Data collection; writing draft manuscript.; b Assistant Lecturer, Department of Community Dentistry, Semmelweis University, Budapest, Hungary. Data collection.; c Assistant Lecturer, Department of Community Dentistry, Semmelweis University, Budapest, Hungary. Proofreading of manuscript.; d Assistant Professor, National Institute of Medical Rehabilitation, Budakeszi, Hungary. Proofreading of manuscript.; e PhD Student, Doctoral School of Mental Health Sciences, Semmelweis University, Hungary, Budapest. Data analysis.; f Associate Professor, Department of Community Dentistry, Semmelweis University, Budapest, Hungary. Supervision of the study.

**Keywords:** dental caries, health behaviour, physical disability, epidemiology

## Abstract

**Purpose:**

The aim of this study was to assess the aetiological factors having an impact on the prevalence of dental caries, missing or filled teeth in a subsample of a single-centre cross-sectional study conducted among the inpatients of the National Institute of Medical Rehabilitation in Hungary.

**Materials and Methods:**

Data collection was carried out through the full mouth screening for dental caries according to World Health Organization (WHO) criteria and a questionnaire covering social background, oral hygiene routine, eating habits for all inpatients who underwent rehabilitation between May 2019 and March 2020.

**Results:**

The mean + standard deviation (SD) DMF-T score in the study sample of 110 physically disabled patients was 18.90 + 7.85. Factors which influenced DMF-T were age, frequency of dental visits and frequency of toothbrushing. The caries prevalence was higher than in the general Hungarian non-disabled population.

**Conclusion:**

In the current study it was apparent that patients with physical disability had less favourable oral health with frequent occurrence of dental caries and missing teeth. Based on the results of the current study, new, targeted prevention and intervention can be developed.

More than one billion people are estimated to live with some form of disability, as discussed in the World Health Organization’s (WHO) world report.^24^ According to the national census of 2011, the total number of people living with disabilities in Hungary was around 500,000, with almost half of them with physical disability (PD) of some extent.^8^ PD results in impaired mobility and increases dependence on caregivers and may also have a negative impact of one’s general and oral hygiene.^19^ Therefore, it is expected that people with disability tend to experience more frequent and more severe oral health problems than their non-disabled peers, thus their oral health implying a direct impact on their general health. This is for particular relevance as better oral health is associated with better quality of life.^3,23^

The effect of PDs primarily manifest on oral health through impaired mobility or low manual dexterity, making tooth cleansing more difficult. Previous studies have indicated that people with PD may not be able to perform the recommended frequency of toothbrushing in order to maintain oral hygiene.^14,21^ This can be exacerbated as some people with PDs may also express inability to chew properly due to paralysis of the facial muscles or problems with movement of the tongue and dysphagia, resulting in food remains in the oral cavity after finishing eating. As food remains in their mouths for a longer period of time, this can set off a cascade leading to tooth decay, gingivitis, periodontitis and mouth infections.^16,18^ Moreover, patients with PDs may also be at greater risk for chronic pain conditions and often experience pain soon after onset of impairments.^5,6^ To manage this chronic pain, they are frequently prescribed painkillers which can conceal symptoms arising from oral diseases as well. Consequently, people with PDs may only consult a dentist at the point of having unbearable pain, usually when conservative dental treatment is unlikely to be possible.

While the rights of people living with disabilities for receiving care are set out by legal acts, there are no regularly collected data on indicators of access to care or whether these legal requirements are actually met. According to the Act No. 26 of 1998 on assuring equal opportunity for persons with disabilities, the particular disability must be taken into account when providing care to improve or prevent the deterioration of one’s health. The act also outlines a holistic approach towards the care of persons with disabilities and regulates the provision of special training of carers. The Hungarian Central Statistical Office reports some data based on the regular censuses of the general population from time to time, yet these reports contain information on the demographics of the disabled, and describe access to social care in broad terms.^25^ As for lower-level regulation, there is no dedicated guidance or clinical pathways available for the management of complex dental care for those with disabilities in the Hungarian healthcare system. In practice, the community-based dental care system should take care of the oral health of people with PDs. According to anecdotal evidence, people with PDs usually visit a dentist within their acquaintance, regardless of the dentist’s formal training or previous experience with such patients. Thus, their limited access to proper, high-quality oral healthcare may be cause for concern. Moreover, some suggest that the type of dental care received is more likely to depend on their disability than their oral health status at the time, whereas people with PDs would require a holistic approach to their oral care.^4,20^

 To the best of our knowledge, there are no data about the oral health of people with PD in Hungary. In 2015, the Department of Community Dentistry, Semmelweis University and The National Institute of Medical Rehabilitation opened a dental office (clinic) to gain comprehensive information on patient’s oral health, health behaviour and dental care undergoing rehabilitation.^15,17^ The National Institute of Medical Rehabilitation is the single largest rehabilitation centre in Hungary, patients can come from any part of the country by referral. Rehabilitation care is provided in 382 beds in nine departments for all ages (Department of Orthopaedic Surgery, Surgical and Rehabilitation Department of Amputations, Rehabilitation Department of Traumatic Injuries, Rehabilitation Department of Spinal Cord Injuries, Rehabilitation Department of Hemiplegics, Rehabilitation Department of Brain Injuries, Department of Bone and Joint Infection and TBC Rehabilitation, Rehabilitation Ward of patients with different disabilities, Department of Psychosomatic and Psychotherapeutic Rehabilitation). Consequently, for the first time, it has been possible to investigate the oral health and treatment needs of a fairly large group of PD patients in Hungary. The aim of this paper is therefore to describe the findings of this investigation, as far as dental caries and the oral health and oral health behaviour of a group of physically disabled people are concerned.

## Materials and Methods

The study was conducted at the National Institute of Rehabilitation, Hungary between 15 May 2019 and 11 March 2020. Data collection was stopped on 11 March 2020, as a national emergency had been declared due the COVID-19 pandemic. The consent of the appropriate ethical committee (Medical Research Council, Hungary, ETT TUKEB IV/1433-1/2020/EKU) was obtained prior to start of the study. In addition, the participants provided written consent to take part. For two patients under 18, their parents/legal guardians gave written consent. Although the data set derived the institutional database did contain personal information on patients (such as their name, address and social security number), these were omitted from the data analysis; therefore, the final analysis data set was anonymous.

### Patients

A total of 110 inpatients have gone through a full dental examination in the study period. Patients with PD were identified through medical chart review by using ICD-10 codes that imply mobility impairment.

### Data Collection

The clinical examination were performed by two calibrated dentists, who assessed the oral mucosa and dental caries. The instruments for oral examination were a plane mouth mirror and a blunt dental probe using artificial light. WHO 2013 criteria were used to record teeth as sound, decayed, missing or filled. Teeth (T) were coded as sound teeth if there was no obvious dental caries or any restoration in the tooth concerned; decayed teeth (D) if there was caries lesion into dentine or filled teeth with decay; filled teeth (F) if there was any kind of filling without obvious decay; missing teeth (M) if it was missing for any reason. Prior to assessing the patients in the sample, the two examiners achieved a Kappa score of 0.81 during calibration.

The questionnaire used in this study included items (Fig 1) on oral hygiene (frequency of toothbrushing, mouthwash usage), health behaviour (eating habits, alcohol and tobacco consumption) and dental care (timing of last visit to a dentist). The questions were based on the recommendations of *Oral Health Surveys: Basic Methods* (5th edition), WHO. Two examiner dentists equipped with tablets assisted the inpatients willing to respond in filling out the questionnaire.

**Fig 1:**
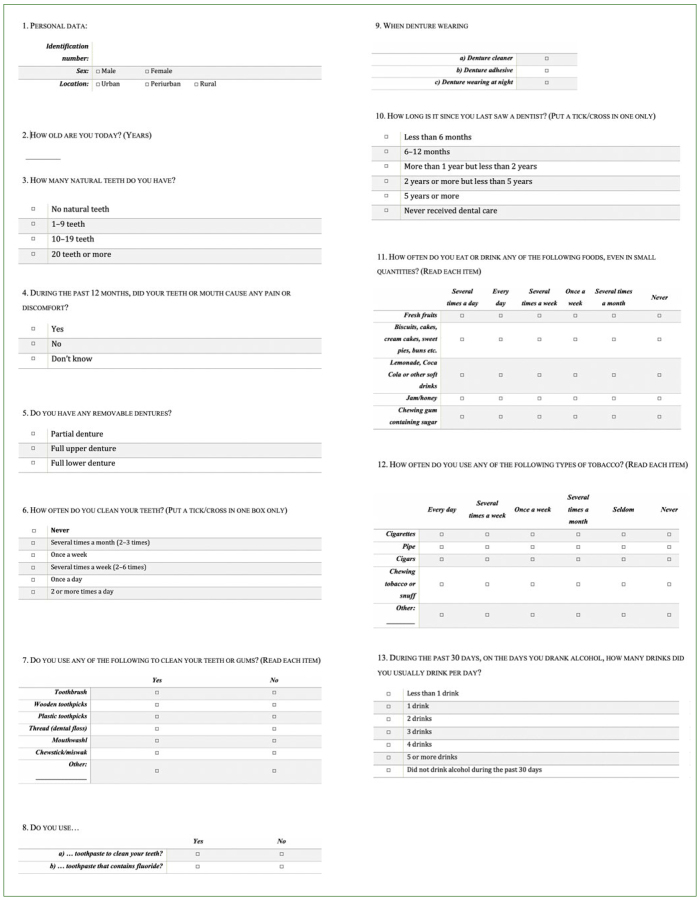
Questionnaire (Part 1) (based on the recommendations of Oral Health Surveys: Basic Methods, 5th edition, WHO).

**Fig 1:**
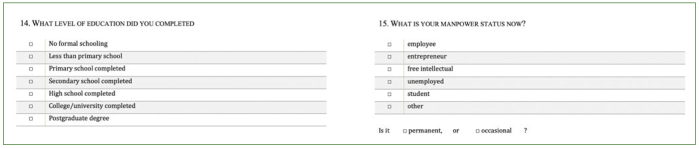
Questionnaire (Part 2) (based on the recommendations of Oral Health Surveys: Basic Methods, 5th edition, WHO).

Basic demographic data such as age, gender, permanent address, employment status and educational level of patients were collected. Level of education level was described as primary (completed primary or elementary school), secondary (high school or vocational school), tertiary (college or university). The Functional Independence Measure (FIM) was used for all patients to evaluate disability and dependency at the start of the rehabilitation. FIM is valid tool for patients undergoing rehabilitation and for the measurement of patients’ functional status and planning treatment protocols. Activities of daily living, which are the purpose of this test, include: self-care, eating, grooming, bathing, dressing, toileting, swallowing, sphincter control, mobility, transfer, and locomotion. Possible FIM scores range from 18 to 126.^22^

### Statistical Analysis

The software and the application for data collection and analysis was created in a collaboration between the research group who performed the study and scientists from the Wigner Research Centre for Physics, Budapest. Data was uploaded directly to the Cloud area allocated to the Hungarian Academy of Science. Statistical analysis was performed using R software (the company based out of 9205 NW 101st St, Miami, Florida, United States). The validity of data entry was checked by randomly selecting observations and checking their entries against the original questionnaires. The descriptive data are presented as frequency, mean, standard deviation (SD) and median.

The total sample size for the group of patients with PD was 110, but some did not answer all the questions in the questionnaire and so the numbers for responses to each question are less than 110 in many cases.

## Results

All of the inpatients were Hungarians. The majority of patients with PDs were males (66 males, 44 females), with a mean age of 53.68 years. In terms of education and occupational status, nearly two-thirds (65.45%) of patients with PD had completed secondary education, and a similar proportion had an active occupation (67.27%). The sociodemographic data and the clinical variables of the patients are summarised in Table 1.

**Table 1:** Sociodemographic characteristics and clinical variables of patients with physical disability (PD), Mean ± SD or n (%)

Variables	Patients with PD (n=110)
Gender	
Male	66 (60%)
Female	44 (40%)
Age; years	53.68 (+18.51)
Education	
Primary	20 (18.18%)
Secondary	72 (65.45%)
Tertiary	18 (16.36%)
Occupational status	
Active	74 (67.27%)
Inactive	36 (32.73%)
Permanent address	
Capital	40 (36.36%)
Other	70 (63.64%)
FIM score*	85.68
DMF-T**	18.90±7.85

*FIM – Functional independence measure. **DMF-T – decayed, missing, filled teeth

The mean DMF-T score of the population was 18.90 ± 7.85. The association of DMF-T; D-T; M-T and F-T with age groups of adults (aged 44 or younger), middle-aged (aged 45–64) and the elderly (aged 65 or older) are summarised in Table 2. DMF-T was observed to increase as age increased among study participants.

**Table 2:** Mean DMF-T; D-T; M-T and F-T by age groups (n = 106)

n	Age	DMF-T	D-T	M-T	F-T
33	– 44	12.79	6.18	2.52	4.09
37	45 – 64	19.22	2.95	9.97	6.19
36	65 –	24.17	2.28	17.69	4.19

As reported with the corresponding D-T, M-T, F-T scores in Table 3, seventy-five (71%) patients had not experienced dental pain in the past 12 months. On the questions concerning dental cleansing habits, sixteen (15%) patients reported brushing their teeth less than daily and 43 (41%) daily. Only 18 (17%) patients with PD had received a full mouth examination in the last 6 months, and a statistically significant proportion (44%) had their last dental visit 24 months or later; D-T scores increased while F-T scores decreased the longer the interval since the last dental examination. Seventeen patients wore a complete denture, 8 (47%) of whom reported using denture cleanser.

**Table 3:** Relation between D-T; M-T; F-T index and independent variables of patients with physical disability (PD), Data are mean (SD) or n

Variables	n (%)	D-T	M-T	F-T
Toothache in the past 12 months (n = 105)				
Reported	30 (28.57%)	5.07 (4.82)	6.77 (7.58)	4.90 (4.93)
Not Reported	75 (71.43%)	3.23 (4.00)	11.76 (10.27)	4.77 (5.06)
Last dental visit ( n = 104)				
≤6 months ago	18 (17.31%)	2.17 (2.90)	10.5 (8.71)	7.94 (5.95)
6-12 months ago	26 (25%)	3.08 (2.88)	5.92 (6.89)	7.42 (5.40)
12-24 months ago	14 (13.46%)	5.43 (6.72)	9.07 (10.56)	2.79 (3.42)
≥24 months ago	46 (44.23%)	4.20 (4.39)	13.28 (10.63)	2.74 (3.24)
Self-reported frequency of tooth brushing ( n = 105)				
Twice or more per day	46 (43.81%)	3.43 (4.43)	8.50 (9.32)	5.70 (5.45)
Daily	43 (40.95%)	3.35 (3.75)	11.35 (10.27)	4.93 (4.80)
Less than daily	16 (15.24%)	5.81 (4.94)	11.38 (8.84)	2.56 (3.42)
Device used for tooth cleansing**( n = 109)				
Toothbrush (electric)	17 (15.60%)	3.76 (4.10)	6.35 (8.18)	6.82 (5.58)
Toothbrush (manual)	93 (85.32%)	3.78 (4.22)	9.34 (9.28)	5.08 (4.95)
Toothbrush (manual or electric) and mouthwash	53 (48.62%)	3.92 (4.41)	7.53 (8.30)	5.98 (5.17)
Toothbrush (manual or electric), mouthwash and dental floss or shiwak or other	10 (9.17%)	1.30 (1.34)	7.70 (8.39)	8.70 (6.43)

As for the frequency of visiting a dentist, patients who had their last dental visit within 6 months had the highest FIM values (97). In terms of frequency of toothbrushing, patients with the lowest FIM values were those who tended to report brushing their teeth less frequently (72). Patients reporting the use of an electric or manual toothbrush and mouthwash in addition tended to have somewhat lower FIM values (98.78) than those who reported the use of only a manual toothbrush (108.47).

The data on self-reported smoking, drinking and eating habits are presented in Table 4. In total, 25% of patients have reported the use of some kind of tobacco product and 23% reported the consumption of alcohol in the past 30 days. A more favourable dental health status can be observed for those who don’t smoke versus those who do, and a similar association is present for those who did not report the consumption of alcohol from the past 30 days versus those who replied otherwise. As for eating habits, the daily consumption of alimentary products with high carbohydrate content was observed alongside an increased M-T index. The number of missing teeth was the highest in patients who reported consuming such products or more times per day; however, the number of patients in this particular subgroup was low (n = 4).

**Table 4:** Self-reported smoking, drinking and eating habits (data reported as frequency, mean (SD)

Characteristic	n (%)	D-T	M-T	F-T
Smoking1 (n=110)				
Yes	27 (24.54%)	5.37 (5.46)	12.48 (11.40)	2.37 (3.61)
No	82 (74.54%)	3.16 (3.68)	9.52 (9.12)	5.71 (5.14)
Alcohol consumption2 (n=102)				
Any other reply	23 (22.55%)	5.33 (5.29)	12.09 (10.91)	3 (4.09)
Not in the past 30 days	79 (77.45%)	3.42 (4.02)	9.94 (9.79)	5.08 (4.99)
Daily consumption of sugar containing food and drink (n = 77) 3				
≥6	4 (5.19%)	0.5 (0.58)	13.75 (13.28)	2.75 (3.77)
5	13 (16.88%)	3.62 (4.68)	10.92 (9.57)	5.23 (4.69)
4	15 (19.48%)	4.46 (5.14)	7.33 (7.23)	5.13 (3.77)
3	14 (18.18%)	3.57 (4.69)	11.92 (11.43)	4.21 (4.56)
≤2	31 (40.26%)	3.45 (4.29)	10.61 (11.09)	4.65 (5.28)

^1^Subcategories of smoking are defined by aggregating the number of patients who reported either the permanent or occasional consumption of any tobacco product (cigarettes, cigars, pipe, snuff or any other kind) ^2^Subcategories of alcohol consumption are created by aggregating the number of patients who reported to regularly consume any amount of alcohol in the past 30 days. ^3^Subcategories of high risk food types are fresh fruit, pies and buns, jam and honey, chewing gum containing sugar, sugar or any kind of sweetener, lemonade or other soft drinks, tea served with sugar and coffee served with sugar.

## Discussion

The findings of the present study have shown that patients with PD have poor oral health in general and high caries prevalence. The DMF-T score observed in the sample of patients was 18.90 ± 7.85, which is widely used indicator of populations’ oral health, and suitable for further comparisons. Lee et al (2019)^12^ found that individuals with disabilities have poorer oral health than the non-disabled, regardless the type of disability. This phenomenon may also be observed in this case as well, as the DMF-T score in the current study was higher than in the local adult population (DMF-T = 16.2) reported by Madléna et al^13^ from a nationwide cross-sectional study, conducted with similar tools. Similar associations were reported between these variables by Pradhan et al 2019.^21^ In the current study, the most important factors that influenced DMF-T were age, frequency of dental visits and toothbrushing.

Interestingly 71% of the patients did not report toothache in the past 12 months which was not reflected in their oral condition. According to post-hoc data collection, this group of patients were frequently prescribed several painkillers during their rehabilitation which as a secondary effect hide their oral complaints, although recall bias may also be present.^5,6^ It is noticeable that the oral hygiene routines might be associated with the degree of self-care and functional independence. Patients with low FIM values had higher usage of mouthwash and electric toothbrushes.

The figures acquired from the data collection on prevalence of smoking and alcohol consumption are similar to the ones observed in national survey, as 26.8% of the Hungarian population reported smoking and 25.4% reported regularly consuming alcohol.^9,10^ However, the observations made in the current study are contrary to what was observed by Glazier et al (2013),^7^ who reported a more prevalent substance abuse among people with disabilities compared to that of people without disabilities. Snacking between meals is also considered as a bad habit and can be a consequence of boredom and hopelessness. In the current study, the daily consumption of high carbohydrate foods was reported with an increased the M-T index. All of the findings in the current study indicated a lack of prevention of oral disease in physically disabled who took part.

For a variety of reasons, the special needs population has difficulty accessing dental care.^11^ Baird et al (2008)^1^ reported that facilities for physically disabled people at general practices are limited. This problem is not just about physical access such as ramps rather than stairs. Even if a PD patient can access a dental office, the dental professionals who care for them are not always trained to manage their care. In Hungary there is no specialisation in special care dentistry and little time is devoted to this aspect during undergraduate training. Dental professionals need to be educated about this subject.

A potential limitation of the current study is that periodontal status was not assessed. This would have provided additional information as these patients can also be hospitalised before starting rehabilitation and this prolonged time can contribute to increased plaque accumulation and gingival inflammation.^2^ The stopping of data collection due the COVID-19 pandemic can be seen as a further limitation of this study, yet we argue that its impact was less statistically significant for institutionalised patients with PD. It has appeared that the restriction and lockdown has not been a life changing situation for people with PD compared to the non-disabled population. However, so far there is no local data yet about the impact of restricted access to dental care on oral health.

Although the sample size of the current study can be considered sufficiently large for descriptive purposes, the validity of the findings for some subgroups can be limited due to low patient counts; further data collection and research is needed to confirm these findings. Also, some observations made in this research (such as the low uptake of electronic toothbrush despite the patients’ need for a caregiver) could be further examined after additional data collection on socioeconomic factors and oral care habits.

## Conclusions

According to this single-centre, cross-sectional study, patients with physical disabilities may experience less favourable oral health status than the general population. The oral health status measured through DMF-T scores, and the prevalence of dental caries and missing teeth can be associated with a number of contributing factors, such as age, frequency of dental visits, oral hygiene routines and health behaviour factors. This information can contribute to designing targeted prevention and intervention models for this subgroup of patients, potentially through the provision of dedicated training to dental professionals to assist the adaptation of patients with physical disabilities to their circumstances.

Oral health is essential to the well-being of those who struggle with health inequalities due to their PD. Access to dental services is key to achieve good oral health, but this can often be limited by factors outside of the physical environment, such as the lack of adequate training. Therefore, it is suggested to perform further studies on the oral health of people with physical disabilities to assess the effectiveness of policy interventions and design evidence-based actions to address their needs.
